# Platelets: the first cellular responders in the foreign body response to blood-contacting biomaterials

**DOI:** 10.3389/fimmu.2026.1758373

**Published:** 2026-03-06

**Authors:** Mackenzie E. Turner, Delaney J. Villarreal, James W. Reinhardt, Christopher K. Breuer

**Affiliations:** 1 The Center for Regenerative Medicine, Abigail Wexner Research Institute Nationwide Children’s Hospital, Columbus, OH, United States; 2 The Ohio State University College of Medicine, Columbus, OH, United States; 3 Department of Surgery, The Ohio State University College of Medicine, Columbus, OH, United States; 4 Department of Surgery, Nationwide Children’s Hospital, Columbus, OH, United States

**Keywords:** foreign body response, inflammation, innate immunity, platelet, tissue engineering

## Abstract

Implanting biomaterials gives rise to the foreign body response (FBR), a complex cascade consisting of blood-material interactions, provisional matrix formation, inflammation, wound healing, and remodeling. While tissue engineering seeks to harness this host response to transform implanted materials into living tissue, the FBR can drive various complications that undermine construct function and longevity with significant clinical impact for patients. The past several decades yielded important insights regarding protein adsorption dynamics and the subsequent cellular responders that exert significant influence over the inflammatory and healing processes governing the FBR. However, the contributions of platelets have often been overlooked and continue to remain underappreciated, especially compared to other major players like macrophages and fibroblasts. Beyond their classical role in hemostasis, platelet-derived products have long been explored for regenerative applications, and platelets are now recognized as immunomodulators. In this review, we highlight platelets as the first cellular responders to biomaterial implantation, emphasizing their active and multifaceted roles in the FBR. We further propose platelet modulation as a strategy to optimize host-material interactions and improve patient outcomes. A complete understanding of the FBR for blood-contacting biomaterials must begin with the arrival of the platelet.

## 1 Introduction

### 1.1 First responders after biomaterial implantation

Cardiovascular disease affects individuals of all ages, from early fetal development through adulthood. Structural anomalies, such as congenital heart defects, along with other vascular pathologies often require surgical repair or replacement of injured, diseased, or absent tissues. These surgical procedures frequently involve the implantation of vascular patches, conduits, stents, or valves - all of which result in the presence of a blood-biomaterial interface. Historically, severe structural anomalies were reconstructed using permanent prosthetic devices composed of insoluble polymers or metals. Complications associated with permanent protheses and their growth constraints when used in children have motivated the search for alternatives.

Over the past few decades, the field of tissue engineering and regenerative medicine has experienced rapid growth, producing a variety of strategies for addressing the limitations of permanent cardiovascular prosthetic devices among other unmet needs in medicine. Tissue engineering employs biomaterials, cells, and/or pharmaceuticals to repair or replace tissue. The success of a tissue engineered construct relies on its biocompatibility, capacity to elicit a suitable host response, and eventual integration with native tissue. Early strategies attempted to grow tissues from a patient’s own cells *in vitro* (1). Safety, regulatory, and cost concerns have spurred interest in *in situ* approaches, whereby implanted materials harness the body’s innate healing capacity to guide neotissue formation. Most biomaterials used in tissue engineered constructs trigger a foreign body response (FBR), characterized by overlapping phases of inflammation, wound healing, and, in the context of tissue engineering, tissue remodeling (1). The nature of the FBR depends on the specific biomaterial, site of implantation, and patient-specific factors. Common to the FBR is the time-dependent infiltration of the implant microenvironment with various immune cell types, including neutrophils and macrophages, that facilitate foreign material degradation and neotissue remodeling (1, 2). The FBR is an essential process in tissue engineered construct integration and from the earliest stages the FBR influences the long-term success or failure of implants. While a hyperactive or prolonged FBR can lead to adverse effects and device failure, completely curbing the host immune response prohibits sufficient neotissue formation and integration of the construct with the surrounding native tissue (3, 4).

For permanent prosthetic devices that contact blood, a critical component of biocompatibility is hemocompatibility, or the relative ability to avoid clinically impactful thrombosis and thromboembolic complications. In contrast, for cardiovascular tissue engineering the absence of thrombosis might not represent the most beneficial host response. Thrombosis can serve important roles, such as facilitating hemostasis in porous constructs or forming a provisional matrix to support neotissue formation (1, 2, 5).

Central to the thrombotic response and resulting thrombus are platelets. Beyond their canonical role in hemostasis, platelets are recently being recognized as significant contributors to immunity (6, 7), wound healing (8–10), tissue remodeling (11), and biomaterial integration (2, 4). The multifunctional nature of platelets has led to recent interest in leveraging platelets and platelet-derived materials in musculoskeletal tissue engineering and wound repair (12). Comparatively, in cardiovascular tissue engineering few studies have intentionally explored the recruitment or utility of platelets and platelet-derived products for improving the outcomes of blood-contacting biomaterials *in vivo*. Instead, the dominant focus has been on preventing platelet activation, adhesion, and aggregation on biomaterial surfaces (13, 14). While uncontrolled thrombosis and thromboembolism necessitate caution, a platelet-avoidant approach often inadvertently overlooks the potential regenerative benefits platelets may confer to engineered constructs.

Platelets are an underappreciated major player in cardiovascular tissue engineering. To date, no comprehensive review has explored their multifaceted role in the FBR within this specific context. This review aims to provide an overview of the crucial, often beneficial, functions of platelets in the FBR to these implanted materials. We focus especially on their interactions with other immune cells and their contributions to tissue remodeling and regeneration. To this end, we highlight the opportunity to strategically modulate the early platelet-mediated events of the FBR for optimizing biocompatibility and regenerative outcomes. Ultimately, we hope this review serves as a resource for investigating and selectively modulating the platelet response to cardiovascular tissue engineered constructs. While previous reviews of the FBR identify neutrophils and macrophages as the first cellular components and influencers of the FBR to vascular biomaterials, we intend to place platelets where they belong: at the beginning.

## 2 Platelets as the assistants of hemostasis: traditional platelet roles

Platelets are small (2-3 µm), anucleate cells within the blood. Upon budding from the megakaryocyte, platelets contain organelles including ribosomes, granules, and mitochondria. These organelles provide platelets with many of the functional capabilities of a typical cell, despite not having a nucleus (15). The platelet plasma membrane is embedded with thousands of receptors that mediate interactions with the surrounding environment (16, 17). Platelets exert their functions directly through these immunoreceptors or indirectly by releasing bioactive molecules stored within their granules and extracellular vesicles (pEVs).

The three major types of platelet granules are alpha, dense, and lysosomal granules. Alpha granules are the most abundant subtype and contain a wide range of cargo including growth factors and other proteins (18, 19). In addition to their contents, alpha granules contain cell adhesion molecules (CAMs) that are translocated to the platelet surface upon granule release. Defective alpha granule secretion impairs platelet spreading on surfaces and limits the release of inflammatory mediators (18, 20). Dense granules, by contrast, house non-protein molecules including serotonin, calcium, adenosine diphosphate (ADP), adenosine triphosphate (ATP), and catecholamines (21). These contents are essential for aggregation, particularly in sustaining aggregation in response to extracellular stimuli. Biogenetically, dense granules are considered lysosome-related organelles (LROs), sharing features with other LROs (e.g., cytolytic granules in T cells) (22, 23). The third type of granules, lysosomal granules, contain proteases and glycosidases, performing degradative functions similar to lysosomes in other cell types.

In addition to granules, pEVs are membrane-bound particles that contain diverse cargo involved in intercellular communication, tissue repair, angiogenesis, and even cancer progression (24). pEVs have significant heterogeneity in their size and cargo, able to carry signaling molecules and cytokines or even organelles like alpha-granules and mitochondria (25, 26) Their functions broaden the physiological role of the platelet and are a relatively new avenue of research.

Traditionally, platelets were thought to interact with the vasculature in three main ways: activation, adhesion, and aggregation. While these terms are conceptually distinct, they occur in parallel and share molecular mediators. Platelets are activated by both pathological and physiological biochemicals, such as coagulation factors, nucleotides, and insoluble proteins. These biochemicals bind to specific receptors with varying affinities and differentially affect platelet function through well-described signaling mechanisms (18). Platelets can also be activated through mechanical stimulation (e.g., shear stress, shear rate) and interactions with artificial surfaces. The placement of tissue engineered constructs in the vasculature alters local hemodynamics and shear forces, thereby creating regions that may promote activation and adhesion (27, 28). Platelet activation initiates a signaling cascade that promotes platelet adhesion and aggregation. This includes overlapping granule release, increased membrane protein expression, and activation of membrane proteins (e.g., GPIIb/IIIa). Upon activation, platelets also express negatively charged phospholipids (i.e., phosphatidylserine) that initiate critical steps of the intrinsic coagulation cascade, culminating in thrombin generation and fibrin polymerization.

Importantly, all mechanisms of platelet activation converge at increasing the likelihood of adhesion and aggregation, typically by potentiating granule release or upregulating adhesion receptors. Adhesion and aggregation are often mediated by the same adhesive ligand and receptor pairs, yet the distinction is based more on the interpretation of experimental studies. Adhesion refers to a platelet transitioning from freely flowing in the blood to arrest on a surface such as the exposed extracellular matrix (ECM) following vascular injury or an implanted material (29). Aggregation refers to platelets binding to other platelets or cells either on a surface or in suspension. Functionally, platelet adhesion to the biomaterial is analogous to primary hemostasis. Aggregation facilitates the formation of a platelet and fibrin-rich mesh that acts as the scaffold for tissue regeneration on the biomaterial surface (1, 30). This is the functional equivalent of secondary hemostasis, or the stabilization of a platelet plug to a thrombus.

## 3 Plasma protein adsorption: calling the first responders

During the initial phase of the FBR, plasma proteins mediate the interaction of cells with a biomaterial surface and serve as a platform for platelet adhesion, immune cell recruitment, as well as the biological processes that follow (1). Biomaterials made from natural ECM proteins (e.g., decellularized cadaveric or *in situ* grown tissue) trigger a response similar to that following vascular injury - where exposed subendothelial collagen and von Willebrand factor facilitate platelet adhesion through GPIV and GP1β, respectively (31, 32). For synthetic biomaterials without a protein component, proteins will rapidly adsorb from the blood upon implantation. Understanding the dynamics of protein adsorption and the subsequent platelet response is important for optimizing the biocompatibility of biomaterials and long-term performance of tissue engineered constructs.

**Figure 1:**
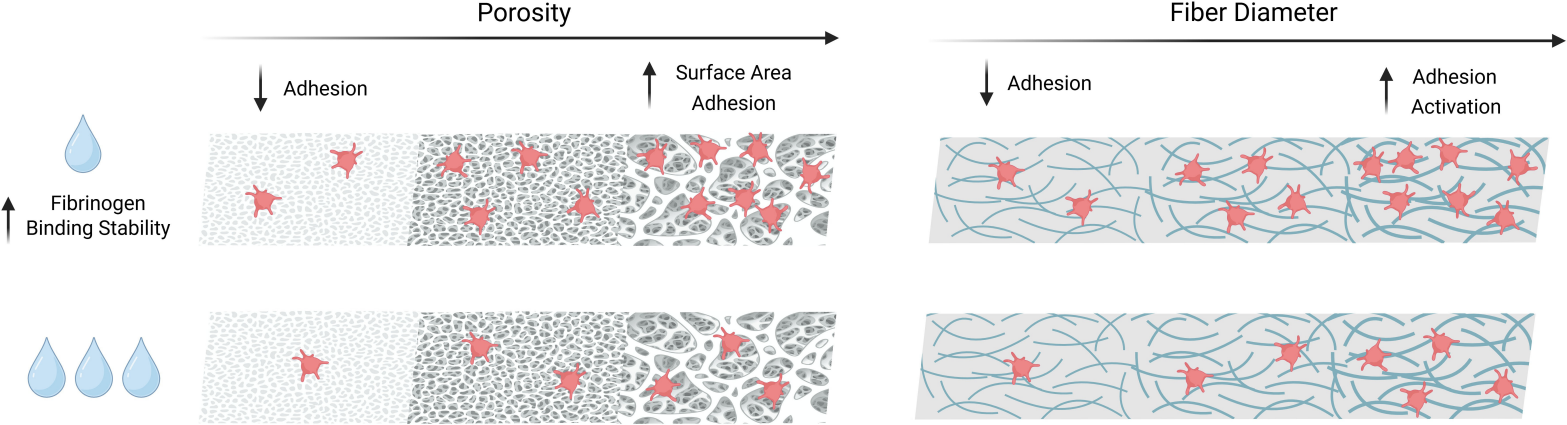
Biomaterial properties known to influence platelet interaction. Simplified schematic summarizing the effects of a biomaterial with low (top) or high (bottom) hydrophilicity along with the conditions of low to high porosity (left) or low to high fiber diameter (right). The level of hydrophobicity has been linked to the stability of fibrinogen binding, influencing the amount of binding substrate for platelet interaction. Higher porosity increases surface area, which also facilitates increased platelet interactions. Lower porosity decreases adhesion, especially when pore sizes are smaller than a platelet. Smaller fiber diameter leads to reduced platelet interactions, and higher fiber diameter allows for more platelet binding. Platelet interaction with a biomaterial is affected by one or more of these factors at once.

The Vroman effect describes the dynamic, time-dependent, competitive displacement of proteins on the surface of a biomaterial, a process governed by the relative abundance of blood proteins and the affinity of the protein for the biomaterial surface (33, 34). Initially, the biomaterial surface acquires a layer of highly mobile and most abundant blood proteins (i.e., albumin and globulin). These proteins have relatively weak affinity for the surface and are quickly displaced by less abundant proteins with a higher affinity. Protein adsorption is influenced by a variety of surface properties, including surface chemistry that may be relatively hydrophilic, hydrophobic, or even oleophobic (e.g., expanded polytetrafluoroethylene (ePTFE)) (**Figure 1**).

Commonly investigated bioresorbable polyesters used in biomedical applications (e.g., PGA, PLA, PCL) are hydrophobic (30). On hydrophobic surfaces, protein adsorption is considered more stable and less reversible, which can enhance the retention of fibrinogen, an important protein for establishing the provisional matrix. However, one study adds nuance to this convention, finding that in addition to hydrophilicity/hydrophobicity, polymer composition also significantly affected the adsorption of fibrinogen. Two distinct polymers possessing virtually identical physicomechanical properties and air-water contact angles differed in fibrinogen adsorption depending on the chemical composition of their surfaces (35). In its native state, fibrinogen does not bind to platelets. Thus, how the biomaterial influences both quantity and conformation of bound fibrinogen (i.e., the reactivity of adhesion domains) is critical for predicting the platelet response. Depending on the chemistry of the blood-contacting biomaterial, fibrinogen may undergo a conformational change that exposes cryptic binding sites for platelet adhesion via GPIIb/IIIa integrin interactions (36). Hydrophilic surfaces acquire less fibrinogen, which can limit thrombogenicity (37). While hydrophilic surfaces may reduce coagulation risks, the lower affinity for fibrinogen may also negatively affect early cell adhesion and integration, a necessary consideration for tissue engineered constructs without an existing endothelial layer (33, 38). The role of other proteins in the adsorbed protein layer (e.g., albumin and high molecular weight kininogen) in platelet-surface interactions is not yet fully understood.

Surface chemistry is an important design parameter for biomaterial selection, as it directly influences protein adsorption, conformation, and subsequent platelet interaction. However, prioritizing a specific surface chemistry could prove overly restrictive, potentially forcing unfavorable trade-offs with other critical material properties, such as degradation rate. Fortunately, there exist post-fabrication strategies for altering material surface chemistry by physical, chemical, and biofunctionalization techniques without affecting bulk material properties (39, 40). The most accessible strategy is physical adsorption which can be achieved simply by incubating a material in solution containing a compound of interest (e.g., heparin) often resulting in short-duration changes to a material’s surface chemistry that dissipate as the adsorbed material becomes unbound. Longer-term changes to a material’s surface chemistry require altering covalent bonds. Chemically adding a high concentration of desired functional groups (e.g., -OH, -NH2, and -COOH) to the surface of polyethylene terephthalate (i.e., Dacron) has been shown to significantly affect the adsorption and denaturation of fibrinogen (41). ECM components covalently linked to ePTFE (i.e., biofunctionalization) reduced platelet adhesion during static incubation with PRP (42). Tropoelastin biofunctionalization reduced the thrombogenicity of polyurethane during static blood incubation *in vitro (*43). Because platelet adhesion and activation are highly sensitive to the identity and structure of the adsorbed protein layer at the biomaterial interface, such surface chemistry modifications provide a non-pharmaceutical approach for altering platelet-mediated initiation of the FBR.

Surface topography and porosity also influence the concentration and spatial patterning of adsorbed proteins (44–47). One *in vitro* study evaluated platelet adhesion and activation on vascular grafts composed of polyesterurethane and poly(lactic-co-glycolic acid) (PLGA) (48). Scaffolds fabricated from each material included smooth, solvent-cast surfaces as well as “rough” surfaces generated by electrospinning, with fiber diameters averaging around 1, 3, and 5 µm. When incubated with heparinized human whole blood, platelet adhesion and activation increased with increasing fibril diameter and were less affected by the chemistry of polymers tested. A separate study compared flat PTFE, ePTFE, and electrospun PTFE with fiber diameters between 0.5 and 3.0 µm, with electrospun PTFE exhibiting less platelet attachment and activation after static incubation with PRP (49). The authors suggested that fibers with diameters smaller than platelets limit spreading, thereby reducing activation.

On fibrinogen-coated surfaces, rough nanofibers resulted in greater platelet surface coverage and spreading during static incubation with PRP in addition to greater accumulation of platelets under flow conditions using whole blood (50). These differences were attributed to higher locally accessible surface area and higher ligand density on nanofiber surfaces compared to smooth planar surfaces. Conversely, despite an increase in surface area, a surface texture consisting of an ordered array of sub-micron pillars restricted the area that a platelet could contact, leading to reduced platelet adhesion compared to a smooth surface (51).

While smooth surfaces present a lower surface area than rough or porous surfaces, which limits the binding opportunities for platelets and leukocytes, this reduced interaction may adversely affect tissue regeneration and neovascularization (45). Adequate porosity and pore size are essential to support tissue integration, facilitate nutrient diffusion, encourage neovascularization, and impact long-term outcomes (44, 46, 47). However, an optimal balance must be achieved between hemocompatibility and tissue regeneration. Protein adsorption is not only significant for platelet adhesion but also impacts the coagulation cascade. Binding and activation of Factor XII stimulates the generation of thrombin through the intrinsic coagulation cascade, contributing to the activation and recruitment of platelets and the production of fibrin, especially in the context of negatively charged surfaces and decellularized material (52). The identity, orientation, and conformational state of adsorbed proteins also modulate whether and how complement activation proceeds, as well as the extent complement components crosstalk with thrombogenic and inflammatory responses. Complement activation is a central driver of the thromboinflammatory response (53). Complement components deposit onto the blood protein layer that forms on the biomaterial surface. Accordingly, the kinetics of protein adsorption, which are shaped by properties such as hydrophobicity, charge, and surface energy, critically influence the mechanisms and potency of complement activation. Importantly, the complement cascade is known to recruit and activate platelets, and conversely, platelets themselves can activate and regulate the complement cascade (54). Given the influence of early hemostatic events on the FBR, understanding these interconnected pathways may reveal opportunities to modulate complement and coagulation to mitigate adverse outcomes.

## 4 Platelets as terraformers of the biomaterial surface

**Figure 2:**
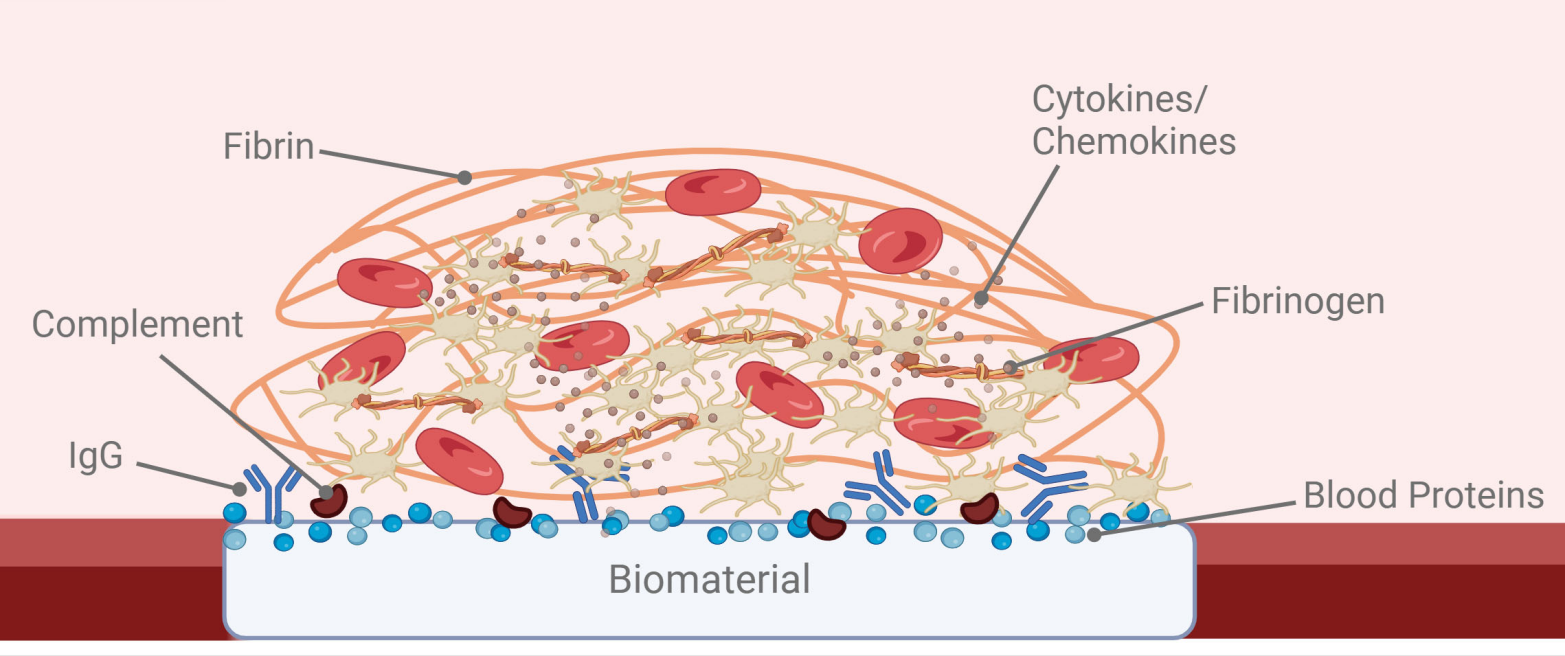
Schematic of the provisional matrix highlighting key components. This schematic represents an illustrative provisional matrix generated on a blood-contacting biomaterial implanted in the vasculature, denoting adsorbed blood proteins, adhesion of circulating complement components, and antibodies. Here also are cytokines and chemokines trapped from circulation by the fibrin mesh or generated by the platelets, red blood cells, and other immune as well as vascular structural cells involved in inflammation and wound healing responses. This microenvironment allows for locally high concentrations of these captured and released factors. As this is an illustrative schematic, the temporal nature of the provisional matrix development was not represented. (Created with BioRender).

The optimal biomaterial design would coordinate protein binding and platelet adhesion to form a relatively thin, non-occlusive, mural thrombus on the surface of the scaffold (**Figure 2**). This provisional matrix, composed of cross-linked fibrin, fibrinogen, platelet-derived growth factors (e.g., TGF-β), and leukocytes, serves as the foundation for cell recruitment, adhesion, and wound healing (30). Granule release from activated platelets leads to the accumulation of locally high concentrations of signaling factors in the provisional matrix. Platelets also contribute factors such as vitronectin and fibronectin that, along with plasma-derived fibrinogen and fibrin, form the initial provisional matrix. One can think of the provisional matrix as a temporary scaffolding and sustained release microenvironment for bioactive molecules involved in tissue repair (1). Many of these platelet-derived molecules act as chemoattractants and cytokines that influence the recruitment and function of other cells responsible for inflammation and remodeling. Other molecules from the platelet releasate are known to be angiogenic factors that help to establish functional and stable vascular networks.

This microenvironment is spatiotemporally dynamic and will change in structure, composition, and mechanical properties throughout the process of neotissue formation and remodeling. As the FBR progresses, the thrombus components are replaced with ECM proteins that promote endothelial attachment and proliferation through a process driven by platelet-derived mediators (e.g., MMPs) and the action of other cells (1). Rapid endothelialization of the luminal surface is important for increasing the long-term hemocompatibility of the tissue engineered construct. Nevertheless, not all thrombi are equal in their ability to promote neotissue formation. Compacted, dense thrombi can restrict cell migration and remodeling as well as prevent transmural angiogenic ingrowth, limiting luminal endothelialization (55). Therefore, the conduciveness of thrombi for promoting *in situ* remodeling depends on the interactions of biomaterial, anatomical location, and other factors. We posit that thrombus properties and provisional matrix formation may be optimized by altering both the material properties and by modulating the immune response.

## 5 Platelets as immunomodulators

Conventionally, reviews focus on macrophages as the initiators and regulators of biomaterial-induced inflammation and healing. More recent reviews position neutrophils as the maestros of repair (56). Herein, we do not seek to refute the critical roles of macrophages and neutrophils but instead to firmly establish platelets as often the first immune cell type to exert significant influence on the FBR. Current perspectives do not adequately recognize platelets for their critical involvement in setting the tone for the inflammatory and remodeling processes that drive biomaterial integration.

The added benefit of platelets will depend upon the specific application. When employing a biologic scaffold, such as a decellularized vessel with relatively low risk for thrombotic complications, platelets do not need to generate the provisional matrix as these scaffolds tend to have a well-established ECM. In this application, the pre-established matrix fulfills the otherwise indispensable role of platelets as terraformers. Their overall impact on the FBR may be less pronounced but will not exclude potential downstream contributions to host cell infiltration, remodeling, and tissue integration. However, a fully synthetic, bioresorbable scaffold relies purely on *in situ* tissue engineering wherein platelets provide the foundation for regeneration.

The success of *in situ* tissue regeneration relies on an effective inflammatory response and endogenous cell recruitment (57) Multiple mechanisms contribute to immune cell recruitment to an implanted construct, including the injured adjacent endothelium, but many of the earliest signals come from the platelets attached to the biomaterial surface that help comprise the provisional matrix (11). The ability of immobilized platelets to recruit other cells involves direct binding interactions and paracrine signaling.

Far from being passive mediators, platelets demonstrate remarkable specificity in their responses (58). While platelets may amplify inflammatory signals in one context, in another they can also modulate the resolution of inflammation. This functional duality underscores their nuanced role in immune regulation. This is particularly important for tissue engineering, for which balancing pro-inflammatory signaling with resolution and repair processes is essential for neotissue formation and physiological remodeling.

**Figure 3:**
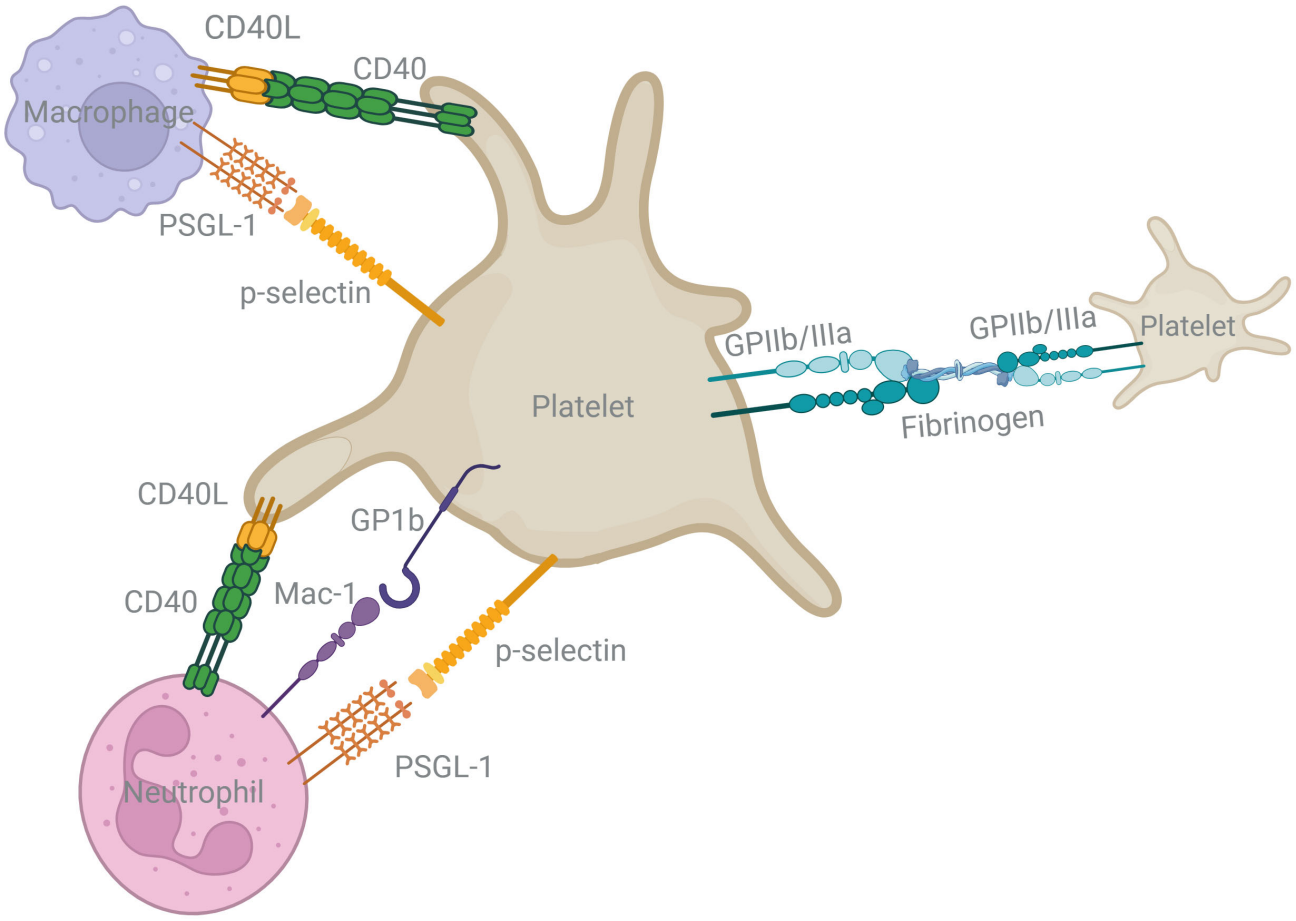
Surface receptor and ligand interactions between platelets and innate immune cells important for platelet immunomodulation during the acute inflammatory phase. This schematic depicts several of the direct binding interactions between platelets and other key innate immune cells in the progression of the FBR to implanted biomaterials. Platelets directly bind and alter the activity of macrophages, neutrophils, and other platelets through several key binding partners including CD40/CD40L, p-selectin/PSGL-1, GP1b/Mac-1, and GPIIb/IIIa binding via fibrinogen. These receptor/ligand pairs are involved in a variety of signaling pathways that influence the phenotype of the target cells.

Despite their small size, platelets possess thousands of cell surface receptors, including numerous distinct CAMs, which facilitate interactions with blood components and other cell types (**Figure 3**). One CAM subtype is a class of transmembrane glycoproteins called integrin receptors, which have diverse physiologic functions important for cell-biomaterial interactions including adhesion, differentiation, and various signaling pathways (59). Platelet GPIIb/IIIa is among the most important integrins for platelet function as it binds immobilized fibrinogen on the biomaterial surface, supporting platelet adhesion, and enables platelet-platelet crosslinking through soluble fibrinogen, resulting in platelet aggregation (60). Platelet-bound fibrinogen and platelet GP1b integrin receptors engage Macrophage-1 Antigen (Mac-1), an integrin expressed by neutrophils, monocytes, and macrophages (61–63). Platelet integrin receptors mediate tethering of leukocytes to the provisional matrix which is particularly important for high shear systems, like that of the arterial environment or the locally high shear sometimes caused by flow disruption from vascular implants (62). In addition, platelet GP1b-Mac1 engagement induces outside-in signaling, contributing to platelet activation (64).

Another essential axis for immune cell recruitment is the interaction between the CAM p-selectin expressed on platelets and p-selectin glycoprotein ligand-1 (PSGL-1) on leukocytes (65). P-selectin/PSGL-1 interaction promotes neutrophil extravasation by mediating capturing and rolling, while also triggering intracellular signals that activate β2 integrins (e.g., LFA-1, Mac-1). These high affinity integrins strengthen adhesion to immobilized fibrinogen, enabling arrest and subsequent cross-talk with immune and non-immune cells (65, 66). This interaction also primes neutrophils for the formation of neutrophil extracellular traps (NETs). NETs, although typically associated with pathogen defense, can form around implanted biomaterials and contribute to a degradative, pro-inflammatory microenvironment that impairs tissue integration (56, 67). Platelets and neutrophils work together to influence acute inflammation and modulate each other’s functions (68). Platelets can promote the release of NETs, and reciprocally, NETs can become a binding substrate for platelets and complement proteins (69, 70). Dysregulation of this initial neutrophil response, including the formation of NETs, can lead to prolonged inflammation around the biomaterial, thrombosis, and has been associated with biomaterial fibrosis (39, 45).

Monocytes are also recruited via p-selectin/PSGL-1. Through this interaction, monocyte-platelet aggregates (MPAs) may form on the surface of the biomaterial (71, 72). Upon p-selectin binding, monocytes upregulate integrins and pro-inflammatory cytokines including monocyte chemotactic protein (MCP-1) and pro-inflammatory tumor necrosis factor alpha (TNF-α) (73–75). While activated platelets may induce and amplify inflammatory signaling, resting platelets also have anti-inflammatory potential, serving to limit inappropriate activation of the immune response. Resting platelets can maintain a resting immunologic state by expressing CD47 to promote monocyte immune quiescence (76). The surface levels of this marker decrease upon platelet activation, thereby unrestricting monocyte responses. In this way, the CD47 interaction limits several key functions driving the progression of the FBR and accordingly, has garnered attention as a potential approach to create immune evasive biomaterials (77–79). Modulating these platelet-immune binding interactions may represent therapeutic targets for influencing downstream FBR signaling.

**Figure 4:**
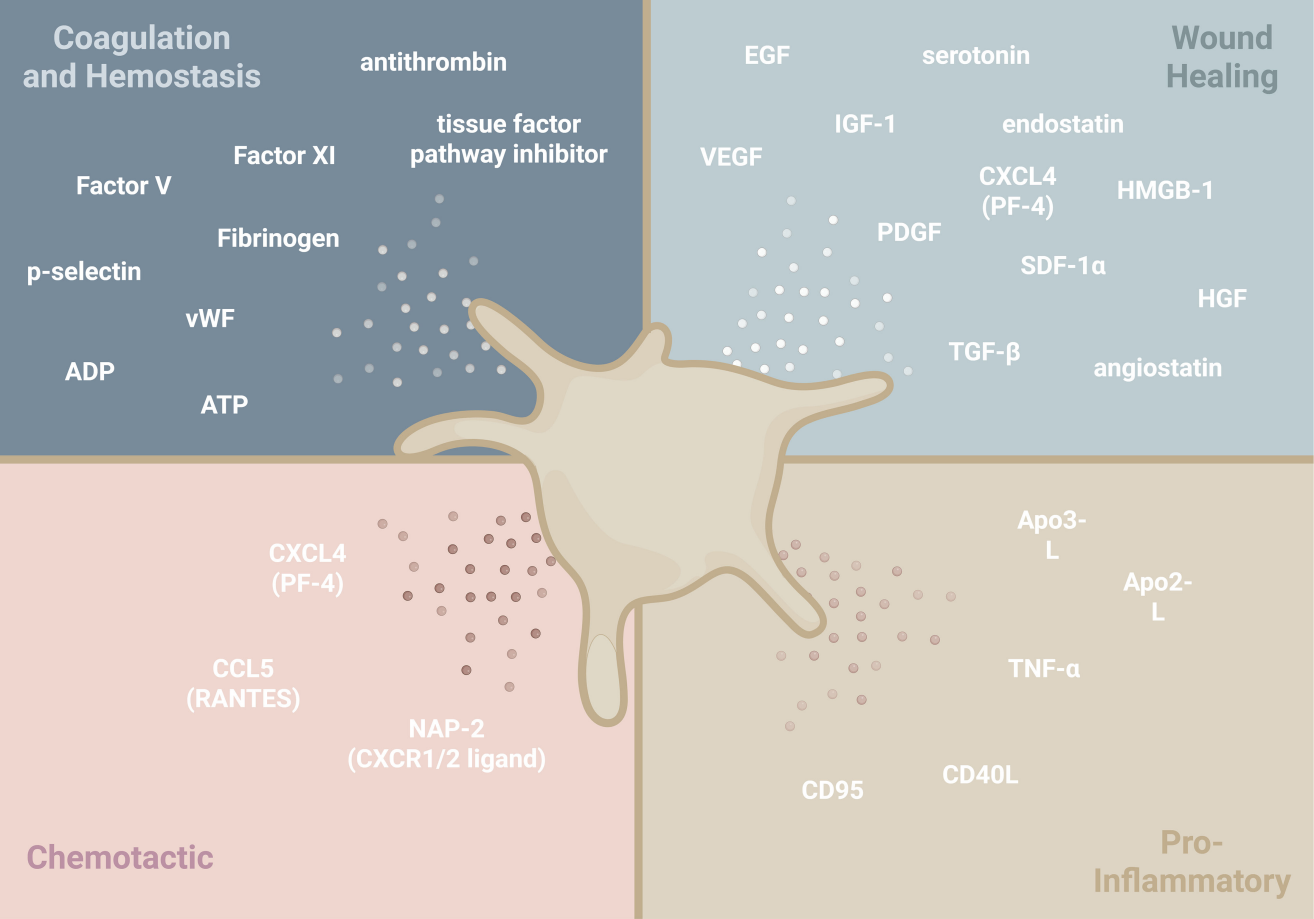
Schematic portraying important platelet secretory factors released during the acute phase of the FBR. This graphic categorizes major platelet-derived soluble mediators into four functional groups: coagulation and injury, wound healing, chemotaxis and cellular recruitment, and inflammation. Adenosine diphosphate (ADP), apo2-ligand (Apo2-L) also known as tumor necrosis factor-related apoptosis-inducing ligand (TRAIL), apo3-ligand (Apo3-L), adenosine triphosphate (ATP), chemokine (C-C motif) ligand 5 (CCL5) also known as regulated on activation, normal T-cell expressed and secreted (RANTES), CD40-ligand (CD40L), chemokine (C-X-C motif) ligand 4 (CXCL4) also known as platelet-factor 4 (PF-4), epidermal growth factor (EGF), hepatocyte growth factor (HGF) also known as scatter factor (SF), high mobility group box 1 (HMGB-1), insulin-like growth factor-1 (IGF-1), neutrophil activating peptide-2 (NAP-2), platelet derived growth factor (PDGF), stromal cell-derived factor 1 (SFD-1α) also known as C-X-C motif chemokine 12 (CXCL-12), transforming growth factor beta (TGF-β), tumor necrosis factor-α (TNF-α), vascular endothelial growth factor (VEGF) also known as vascular permeability factor (VPF), von Willebrand factor (vWF).

Beyond direct binding between platelets and leukocytes, the platelet secretory repertoire is extensive, encompassing over 300 proteins and small molecules (58). A diverse range of chemokines, cytokines, transcription factors, and other signaling molecules are released from the platelet granules or vesicles (**Figure 4**). These mediators are thoroughly described in the context of inflammation. As the FBR is a careful balance of initiating and resolving inflammation, it is important to address how platelets change their secretory behavior to finely tune the microenvironment of an implanted biomaterial. While the participating molecular machinery involved in granule release is cleverly described by Joshi and Whiteheart, the precise mechanisms underlying platelet exocytosis remain incompletely understood (80). Emerging research suggests that platelets release contextually appropriate cargo from alpha granules, a concept that may be necessary to consider when designing blood-contacting biomaterials to optimize platelet responses (19). The field offers two mechanisms supporting controlled platelet granule release: 1.) differential packaging of granules and 2.) differential release of these granules.

The extent of granule release is also influenced by the spatiotemporal nature of the interactions of platelets with the biomaterial and developing neotissue microenvironment. The quantity of infiltrating platelets, size of the developing thrombus, and residence time at the biomaterial interface can affect the nature of the platelet response. Additionally, agonist potency influences kinetics and extent of secretion. ADP, for example, is a “weak” agonist because while it induces aggregation, on its own, it does not stimulate dense granule release. However, ADP enhances the responsiveness of platelets to other agonists like thrombin (81, 82). On the other hand, “strong” agonists (e.g., thrombin and collagen) at sufficient concentrations may stimulate aggregation and dense granule release. Ultimately, addressing the questions surrounding the selectivity of granule release may allow for specific manipulation at the immune microenvironment without interfering with the processes necessary for implant integration with host tissue.

## 6 Platelets as drivers of vascular biomaterial integration: lessons learned from angiogenesis

This section aims to discuss recent progress in connecting platelets to biomaterial-induced tissue regeneration. Many of the original concepts are derived from wound healing studies and investigations of aberrant angiogenesis in a range of pathological conditions, including cancer and atherosclerosis. In the context of *in situ* tissue engineering, as many scaffolds and decellularized tissues begin avascular, the success of any tissue engineered construct of appreciable thickness requires vascular support. Vascularization processes serve two primary purposes: supplying the developing tissue with oxygen via the formation of microvasculature and aiding neovessel remodeling by providing an avenue for localized leukocyte recruitment from the circulation within the developing tissue. This review paper is not the first to discuss the importance of platelets and their granular cargo in the regulation of angiogenesis. Platelet-derived molecules and materials are already applied in tissue engineering and regenerative medicine (10); however, the dynamic interplay between platelets and angiogenic processes remains poorly understood, particularly in the context of implanted biomaterials. In addition to vascularization, luminal endothelialization is critical for long-term outcomes of blood-contacting biomaterials by creating an anti-thrombogenic surface. The mechanism by which luminal endothelization occurs continues to be a subject of debate. Ingrowth from the endothelium of the adjacent tissue, transmural migration (i.e., angiogenesis), and fallout endothelialization from the circulation have all been proposed and are supported by experimental observations (83–85). The relative contribution of each likely depends on the scaffold design and anatomical location. While the effect of platelets will be discussed in the context of angiogenesis, similar mechanisms promote luminal endothelization through chemotactic and pro-mitotic signaling as well as the recruitment of supporting cells (i.e., SMCs, pericytes).

Platelet adhesion to endothelial cells and ECM components has proven critical for angiogenesis and preventing hemorrhage from developing neovessels (86, 87). Both treatment with anti-platelet antibodies (i.e., platelet depletion) and GP1b deficiency individually prevented platelet receptor binding to von Willebrand Factor and thrombin. In both scenarios, preventing platelet adhesion led to dysregulated angiogenic processes and the generation of unstable blood vessels in mice, with platelet depleted mice having a more severe phenotype (86). While neovascularization still occurred in the absence of platelet adhesion, there was significant hemorrhage through the newly formed vessels. Defective dense granule exocytosis did not affect the formation of stable angiogenic vessels. Recall that dense granule secretion promotes sustained aggregation but is not required for adhesion. Consistent with this notion, defective dense granule exocytosis did not adversely affect neotissue formation in tissue engineered vascular grafts (4). Collectively, these findings suggest that platelet adhesion is critical to support tissue regeneration around implanted scaffolds.

The platelet membrane itself also supports angiogenesis with components such as sphingosine-1-phosphate (S1P) and phosphatidic acid, which enable haptotaxis for endothelial and smooth muscle cells (SMCs) that eventually comprise the neovessel. The provisional matrix contains numerous alpha granule-derived growth factors, namely vascular endothelial growth factor (VEGF) (88–90), platelet-derived growth factor (PDGF) (91), basic fibroblast growth factor (bFGF) (92, 93), epidermal growth factor (EGF) (94, 95), transforming growth factor beta (TGF-β) (96), insulin-like growth factors (IGF) (97), and angiopoietins (Ang). The PDGF, FGF, VEGF, and Ang1 signaling pathways have garnered attention for their influence on wound healing and blood vessel formation. Myeloid cell-derived PDGF-B was shown to regulate intimal neotissue development on tissue engineered vascular grafts, with myeloid-specific PDGF-KO mice developing less neotissue, decreased SMC proliferation, and decreased collagen content (98). This study did not specifically investigate platelets, yet this suggests that PDGF released by platelets may contribute to vascular neotissue formation by promoting vascular SMC and myofibroblast proliferation and secretion of ECM constituents.

Platelets also carry angiogenesis inhibitors in their alpha granules. The concurrent release of inhibitory factors with pro-angiogenic factors ensures that vascularization occurs in a controlled and organized manner, limiting aberrant remodeling processes (e.g., neointimal hyperplasia, fibrosis) that might impair graft function or stability. This intrinsic regulatory mechanism contributes to the fine-tuned vascular response to implanted biomaterials that, when balanced appropriately, ensures proper tissue remodeling and functional integration. Well-described platelet-derived suppressors of angiogenesis include endostatin, angiostatin, and platelet-factor 4 (PF4), all of which interfere with growth factor signaling to reduce migration and proliferation of endothelial cells (99, 100). Endostatin has been utilized in cartilage engineering to inhibit vascularization of engineered constructs (101). These inhibitory pathways may also be modulated for optimizing angiogenic outcomes in tissue engineered constructs. For example, localized release of endostatin or angiostatin may be favorable for modulating excessive angiogenic responses.

## 7 Platelets as a target for modulating the FBR: existing pharmaceutical strategies

### 7.1 P2Y12 inhibition

**Figure 5:**
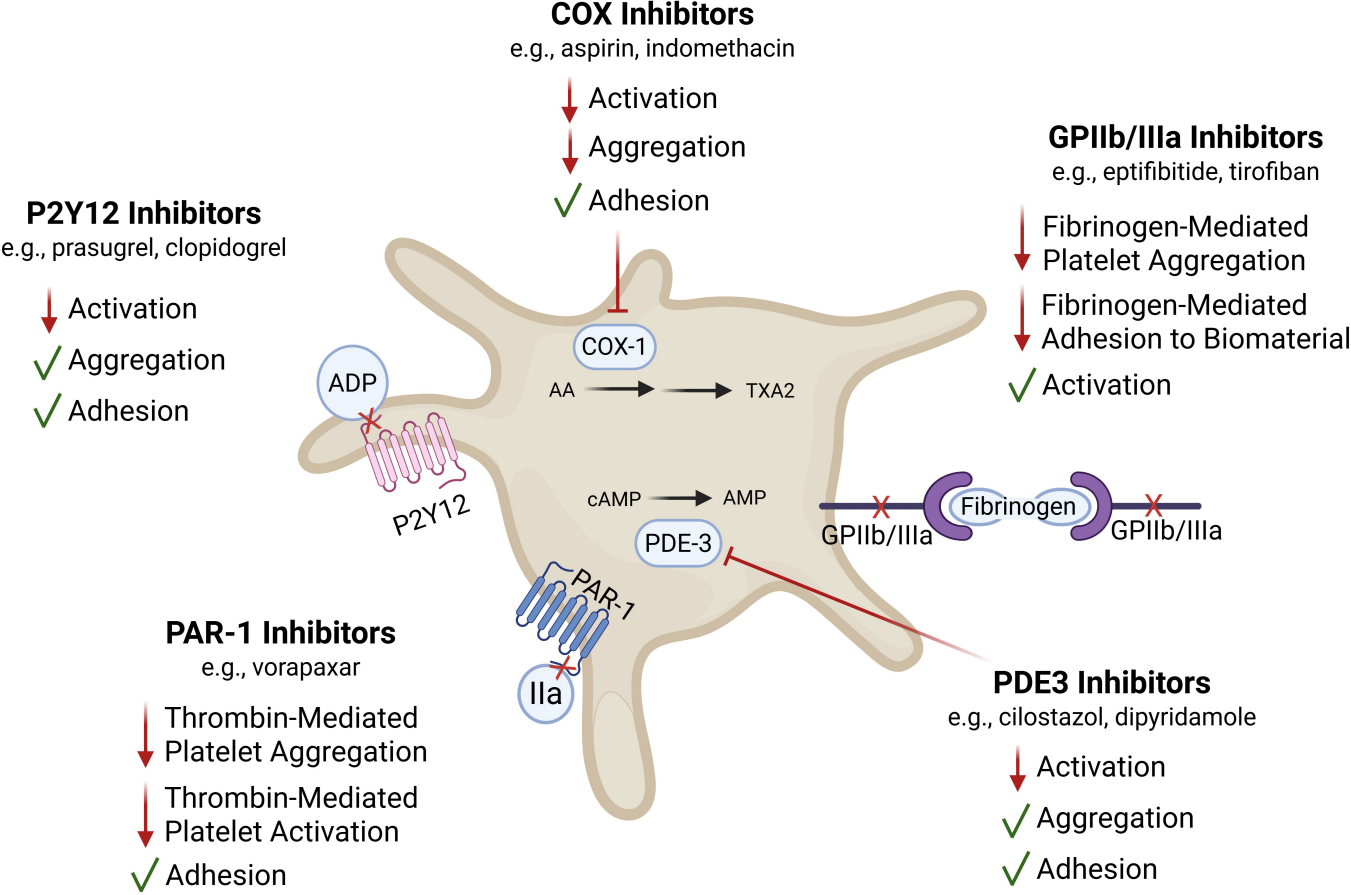
Selected pharmacological strategies for targeting platelet function that influence key events in the FBR. This figure depicts key anti-platelet pharmaceutical strategies and summarizes how their primary mechanism of action impacts platelet activation, aggregation, and adhesion. Understanding that these anti-platelet medications often affect one aspect of functionality while preserving others enables intentional selection of an anti-platelet medication to allow for modulation of the platelet response without complete inhibition. The inside of the central platelet depicts a simplified mechanism showing how these medications affect intracellular signaling pathways and subsequent platelet functions. COX-1 mediates thromboxane A2 (TXA2) generation, and TXA2 amplifies platelet activation through Ca^2+^-dependent signaling pathways that promote secretion and integrin activation. In contrast, cAMP is a major inhibitory second messenger, which activates PKA, suppressing Ca^2+^ mobilization and downstream activation pathways. PDE3 inhibitors decrease intracellular cAMP by limiting its degradation, thereby reinforcing this inhibitory axis. Arachidonic acid (AA), adenosine diphosphate (ADP), adenosine monophosphate (AMP), cyclic adenosine monophosphate (cAMP), cyclooxygenase-1 (COX-1), glycoprotein IIb/IIIa (GPIIb/IIIa; integrin αIIbβ3), phosphodiesterase-3 (PDE3), proteinase-activated receptor 1 (PAR-1), and thromboxane A2 (TXA2).

P2Y12 represents one of the many P2Y/P2X receptors that exist on platelets. Collectively, this class of receptors contributes to platelet activation and aggregation through purinergic signaling. ADP binds to the P2Y12 receptor, which potentiates agonist-induced granule secretion, recruits platelets and inflammatory cells, and promotes thrombus growth and stability, reinforcing aggregation at the edge of a developing thrombus (102–104) (**Figure 5**). ADP-mediated signaling through P2Y12 does not affect adhesion of platelets to fibrinogen and collagen, two substrates common at the biomaterial interface (102, 103). Thus, modulating ADP-mediated platelet aggregation appears to be a viable strategy for preventing excessive platelet signaling while preserving necessary adhesion processes. In support of this concept, P2Y12 inhibition by clopidogrel and prasugrel treatment reduced platelet-driven aberrant remodeling of small diameter tissue engineered vascular grafts (4, 105). The authors proposed a model wherein early platelet aggregation controls the size of the developing thrombus/provisional matrix on the luminal surface of a bioresorbable scaffold (4).

Separate from direct effects on platelet aggregation and thrombus size, P2Y12 signaling also contributes to the platelet pro-inflammatory processes and platelet-leukocyte aggregation, suggesting that P2Y12 inhibitors could serve anti-inflammatory purposes (106, 107). Evidence suggests that monocytes (108), macrophages (109), T lymphocytes (110), neutrophils (111), and endothelial cells (112) also express functional P2Y12 receptors. While the functional relevance of P2Y12 on other immune cells is unclear, P2Y12 may play a broader role beyond aggregation, as P2Y12 signaling has been shown to promote cell migration (112). There are various FDA-approved P2Y12 inhibitors with considerably safe side effect profiles. Thus, P2Y12 inhibition has the potential to reduce thrombotic complications without adversely affecting wound healing and regeneration, making it advantageous for use with implanted vascular biomaterials.

### 7.2 Cyclooxygenase inhibition

Aspirin has long been a mainstay platelet modulator (**Figure 5**). Several regenerative medicine applications use aspirin to prevent thrombosis after implantation of blood-contacting biomaterials. Despite this intention, aspirin alone may not be sufficient to prevent thrombosis of vascular biomaterials (113). Aspirin has partially selective activities on platelet functions, as it prevents thromboxane (TXA_2_) production and therefore TXA_2_-mediated platelet activation, leaving signaling in response to ADP and thrombin largely intact (114). Beyond its platelet activation-related impacts, it also has several anti-inflammatory effects (115). It decreases the release of several platelet-derived factors and attenuates platelet-driven leukocyte activation and aggregate formation (116).

### 7.3 GPIIb/IIIa receptor inhibition

GPIIb/IIIa is a major platelet integrin responsible for fibrinogen-mediated platelet aggregation. There are numerous clinically approved inhibitors of GPIIb/IIIa, including abciximab, eptifibatide, and tirofiban. Newer second-generation agents such as zalunfiban are under clinical evaluation and may offer improved safety profiles. Direct inhibitors of the GPIIb/IIIa receptor prevent platelet-platelet interactions mediated by fibrinogen (117) (**Figure 5**). Additional effects include anti-inflammatory activity by blocking IL-1β synthesis, reducing platelet-leukocyte aggregate interactions, and blocking sCD40L release (118). However, it is important to note that subclinical doses of these antagonists actually induce a pro-inflammatory state by increasing p-selectin expression, leading to increased platelet-leukocyte aggregates and CD40L release (118). Unlike P2Y12 inhibitors, GPIIb/IIIa receptor inhibitors may also affect platelet interactions with a biomaterial surface or decellularized scaffold, because they prevent platelet binding with adsorbed fibrinogen. Therefore, the use of GPIIb/IIIa inhibitors should be carefully considered because of their impact on the adhesion processes involved in hemostasis and the potential to prevent platelet accumulation in the provisional matrix with downstream impacts on neotissue formation.

### 7.4 Protease-activated receptor-1 inhibition

Thrombin is a strong platelet agonist acting through protease-activated receptors (PAR) that generally results in platelet activation and robust granule secretion (**Figure 5**). Thrombin is also a pro-coagulant that catalyzes the polymerization of soluble fibrinogen to insoluble fibrin. Thrombin produced at injured sites contributes to irreversible platelet aggregation in the core of a thrombus and dense granule secretion, which is responsible for platelet recruitment at the luminal surface (i.e., shell) of the thrombus (119). Vorapaxar is a competitive antagonist of the PAR-1 receptor (120). Inhibiting PAR-1-mediated platelet activation reduces aggregation without affecting platelet-fibrin clot characteristics, coagulation profiles, or platelet aggregation stimulated by ADP or collagen (121). This suggests that vorapaxar may be used to attenuate platelet aggregation responses and thrombus growth without disrupting all platelet activity.

Similar to other classes of antiplatelet drugs, PAR-1 inhibitors demonstrate anti-inflammatory properties. In addition to platelets, endothelial cells and certain leukocytes express functional PAR receptors, consistent with PAR inhibitors affecting diverse cellular activities related to vascular integrity (122). In studies with both animal models and human subjects, vorapaxar consistently decreased expression of circulating pro-inflammatory cytokines (123). Importantly, vorapaxar treatment also decreased infiltration of macrophages and T cells to areas of vascular inflammation (122). Therefore, the off-target effects must be noted when considering using this antiplatelet drug class in the context of biomaterials.

### 7.5 Phosphodiesterase type 3 inhibition

PDE3 inhibition prevents platelet activation caused by stimuli such as collagen, ADP, arachidonic acid, adrenaline, and shear-induced activation (124) (**Figure 5**). Treatment with the PDE3 inhibitor, cilostazol, decreased platelet release of several immunomodulatory and regenerative molecules, namely p-selectin, PF4, and PDGF (124). Due to its short half-life, platelet function recovers rapidly after treatment, suggesting its utility for short-term regulation of platelet-mediated inflammation (125). An *in vivo* study revealed that cilostazol had protective effects against bioabsorbable graft stenosis possibly by preventing platelet activation and attachment to the graft and/or by modulating the acute FBR (113).

Determining the optimal degree of attenuation to reduce excessive platelet aggregation while maintaining adequate provisional matrix formation will require dedicated experimental studies for each biomaterial application. To determine the best pharmaceutical intervention, one must delineate the material- and application-specific platelet signaling pathways that drive the platelet responses (**Table 1**).

**Table 1:** Platelet-targeting pharmaceutical strategies.

Drug class	Primary target/mechanism of action	Effect on platelet activation	Effect on platelet aggregation	Effect on platelet adhesion	Relevance to tissue engineering	Citations
P2Y12 Inhibitors	Block ADP-mediated platelet aggregation	↓ Activation	↓ Aggregation	Preserved	Demonstrated relevance to TEVGs, where aggregation potentiated by ADP contributes to stenosis; allows for platelet adhesion while limiting further aggregation and thrombus stabilization	(4, 102–112)
COX Inhibitors	Inhibit COX-1, leading to decreased TXA_2_ production	↓ Activation	↓ Aggregation	Largely preserved	Reduces early platelet activation without abolishing surface adhesion; may allow for initial platelet-material interactions needed for neotissue formation while limiting occlusive thrombosis or excessive provisional matrix formation	(113–116)
GPIIb/IIIa	Block fibrinogen binding to GPIIb/IIIa	Preserved; Paradoxically ↑ Activation	↓↓ Aggregation	↓ Fibrinogen-mediated adhesion	Potentially prevents fibrinogen-mediated platelet-platelet and platelet-biomaterial bridging; useful for acute thrombosis prevention but may impair potential pro-regenerative platelet functions	(117, 118)
PAR-1 Inhibitors	Block thrombin-mediated platelet activation	↓ Activation	↓ Aggregation	Preserved	Selectively inhibits thrombin signaling, a major driver of pathological thrombosis, while maintaining early adhesive interactions that may facilitate regeneration	(119–123)
PDE3 Inhibitors	↑ cAMP via PDE3 inhibition	↓ Activation	↓ Aggregation	Preserved	Elevated cAMP dampens platelet reactivity broadly; has been associated with improved patency of TEVGs and may promote a less inflammatory remodeling environment	(113, 124, 125)

## 8 Platelets as facilitators of long-term outcomes

Increasing evidence connects the earliest platelet responses and provisional matrix composition with measures of long-term biomaterial performance (i.e., fibrosis and calcification) (126). Platelets have been associated with both promotion and inhibition of fibrotic processes. Notably, platelets significantly contribute to circulating TGF-β levels and regulation of its activation state. TGF-β1 signaling initiates collagen accumulation, which is important to healing but, in excess, can tip the balance toward fibrosis (127). Increased platelet infiltration and therefore increased local concentrations of platelet-derived growth factors, may drive endothelial (128), fibroblast (129–131), macrophage (126), and SMC (132) chemotaxis and phenotypic transitions contributing to fibrosis. One study found that platelet depletion decreased thrombus fibrosis (133); however, completely removing platelets may have unintended consequences such as hemorrhage or impaired neotissue formation. Attenuating certain signaling pathways without completely removing platelets may mitigate undesired fibrosis while allowing for platelet-mediated healing. Nevertheless, platelets are just one of the many cells at the gateway to fibrosis. Thus, future mechanistic studies are necessary to fully elucidate how platelets and other cell types coordinate fibrosis in the context of implanted materials.

Additionally, platelet activity has been connected to biomaterial calcification. Several groups synthesized the potential mechanisms through which platelet function and releasate are connected to calcification. To briefly summarize, platelets can influence oxidative stress and release various bioactive molecules (e.g., osteocalcin (134), EVs, microparticles (135)) that can affect the phenotype and behavior of vascular cells. There remains little to no experimental evidence supporting causative relationships. Nonetheless, the potential for platelets to set the stage for calcification is not a baseless concept. Higher platelet reactivity correlated with a higher arterial calcium burden (136), while platelet depletion seemed to protect against biomaterial calcification (121). This connection is not surprising considering how intimately platelets are involved with inflammation and regulation of vascular cell phenotypes. Despite mechanistic implications, there remains no consensus on the pathology of biomaterial calcification and no clear method to separate the impact of platelet signaling mechanisms from other inflammatory processes.

## 9 A spotlight on platelets in tissue engineering and regenerative medicine

**Figure 6:**
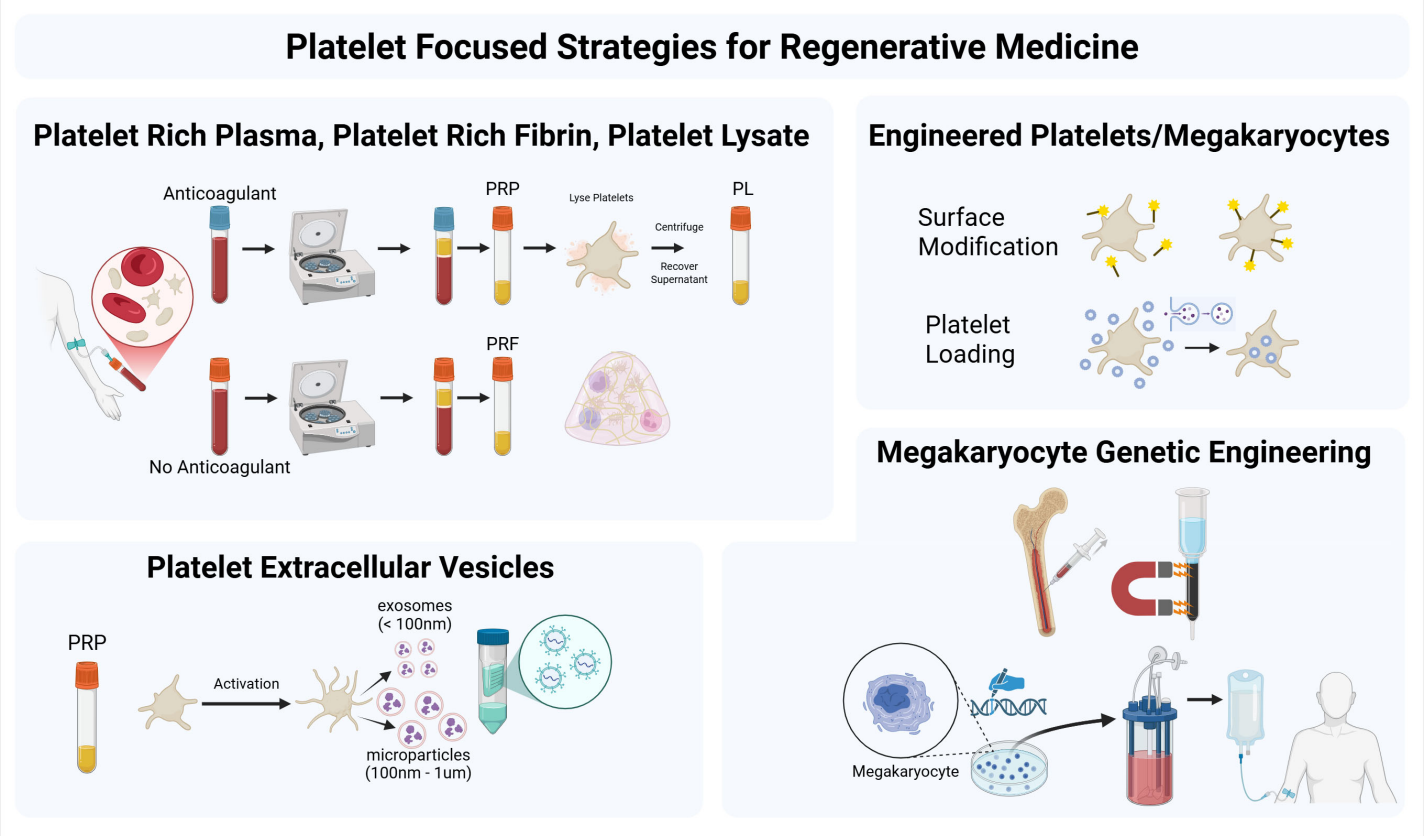
Platelets and their derivatives in regenerative medicine. Schematic overview of platelet-derived approaches used or proposed for regenerative medicine applications. Platelet-rich plasma (PRP), platelet-rich fibrin (PRF), and platelet lysate (PL) represent clinically used platelet-derived products that deliver growth factors and cytokines to support tissue repair. Platelet extracellular vesicles (pEVs), including microparticles and exosomes, provide a cell-free modality capable of transferring platelet-associated proteins, lipids, and regulatory RNAs to recipient cells. Next-generation strategies include engineered platelets generated through surface modification or cargo loading, as well as genetic engineering of megakaryocytes to produce platelets with tailored surface phenotype and defined cargo packaging. These platelet-based and platelet-derived approaches highlight opportunities to harness platelet regenerative signaling and, in principle, tune early platelet-mediated interactions relevant to blood-contacting biomaterials and downstream foreign body response pathways.

The field of cardiovascular tissue engineering and regenerative medicine historically held the objective of minimizing the presence and activity of platelets due to concerns regarding thrombosis. Some groups have attempted to curb the otherwise immediate adsorption of blood proteins and binding of platelets altogether by fully endothelializing the scaffold prior to implantation (137). Novel investigations reveal that some degree of platelet interaction with a biomaterial can be beneficial. The field now looks towards strategies for platelet and FBR attenuation rather than complete inhibition. The overarching strategies include pharmaceutical interventions (Section VII), engineered biomaterials (i.e., drug-elution or inherently modulatory materials such as fibrin), as well as employing platelet-derivatives (**Figure 6**).

With the goal of curbing excessive platelet activation and subsequent cascading inflammatory responses, some implanted blood-contacting biomaterials are synthesized to elute various anti-thrombotic and anti-platelet agents. The most common drugs investigated are heparin (138–141), and aspirin or aspirin-derivatives (142–144). Depending on duration of drug-elution, these may be an option to prevent thromboembolic complications in applications with long-term risk for thrombosis. Importantly, for these drug-eluting devices one must ensure that the agent does not fully inhibit platelet adhesion. While drug-elution is not permanent, more short-acting pharmaceutical intervention may be advantageous for local and temporally restricted control of the acute FBR. 

The therapeutic effects of platelets can be achieved not only through the recruitment of platelets *in vivo*, but through designing scaffolds that incorporate platelet-derived products to enable greater control over neotissue formation. Namely, these products include platelet-rich plasma (PRP), platelet-rich fibrin (PRF), platelet lysate or releasate (PL), pEVs, and engineered platelets. Platelet products come in different physical forms (e.g., gels, liquids, conjugates) and contain diverse mixtures of growth factors and cytokines (145–148). PRP is enriched from whole blood by centrifugation and contains a concentrate of platelets in plasma. Plasma is distinct from serum in that serum is made from whole blood or PRP following natural or induced coagulation. Therefore, serum is plasma depleted of fibrinogen, platelets, and other clotting factors and will contain platelet releasate. Plasma retains the ability to coagulate, a property that can be advantaged to form gels. PRP can be applied on its own or used as a liquid carrier and pro-angiogenic cell delivery system (149). PRP has been used to seed decellularized heart valves and scaffolds, improving their biocompatibility and functionality (150). PRF is a modification of PRP, similarly obtained by centrifugation of whole blood (151). PRF may be prepared as a liquid or a gel, differing from PRP in that fibrinogen has been polymerized into fibrin, there is a higher concentration of leukocytes, and it does not require thrombin for gelation. PL is obtained by disrupting platelets so that their contents are released, commonly through a freeze-thaw process, followed by filtration to remove debris and other unwanted substances.

PRP, PRF and PL are minimally manipulated, autologous products that can be readily prepared at the point-of-care, and do not carry a risk of transmitting infectious agents, lowering the risks to a patient. Therefore, platelet-derived products may be advantageous to tissue engineering, allowing for more fine-tuned control over local concentrations (i.e., of platelets and their cargo) and release profiles to adjust the regenerative microenvironment for a specific application. This is especially true for blood-contacting biomaterials. Platelet-derived materials can be used to pre-clot the implant, both decreasing permeability of blood and fluid through the polymer as well as preventing excessive clotting activities and controlling thrombus size. Several of these materials can also be used as carriers for cells other than platelets to adjust the local microenvironment and promote wound healing and angiogenesis pathways. A caveat to the design of scaffolds incorporating platelet-derived materials is that these would likely constitute a combination product through the eyes of the FDA which would increase the regulatory burden surrounding their clinical implementation in the United States, as compared to either a scaffold (i.e., device), or platelet product (i.e., biologic) used on its own.

Requiring more advanced methods for isolation and manufacture are pEVs that carry a variety of bioactive molecules and engineered platelets (152). pEVs include both exosomes (<100 nm) or microparticles (100 nm to 1um) based on size, which differ in biogenesis (endosomal versus plasma membrane shedding). pEVs carry regulatory RNA, proteins, and lipids which can influence recipient cells through cargo transfer and functional surface receptors, enabling effects relevant to regeneration such as angiogenic signaling, cytoprotection, and immune modulation (153). Compared to PRP, pEVs offer practical advantages including a higher concentration of growth factors, reduced immunogenicity, and improved transport across biological barriers due to their small size (154). To date, clinical translation of pEVs has been demonstrated in a first-in-human phase I clinical trial for the treatment of delayed wound healing(155). There have not yet been clinical applications of pEVs specifically in blood-contacting biomaterials, but there are several examples of EV-loaded scaffolds in early stages of development(156). An important caveat for blood-contacting biomaterial applications is that pEVs can exhibit greater procoagulant activity per unit of surface area than activated platelets, attributed in part to enrichment of phosphatidylserine and membrane-associated coagulation proteins during vesicle shedding(157). Thus, while pEVs may support regenerative signaling, they may also pose a thrombogenic risk in blood-contacting biomaterial contexts and therefore require careful design and evaluation.

Engineered platelets may take a number of forms and can be selectively modified to promote the release of certain factors or possess altered surface properties (144). Platelets may be drug-loaded by platelet phagocytosis, with storage in alpha granules for future release (158). Platelet surface modification can also be used to tether platelets to a therapeutic cell type for delivery to a site of injury or wound healing using the platelets’ innate homing specificity (159). Genetically engineered platelets may be generated through manipulation of megakaryocytes to alter granule content and surface displayed proteins (160) (161).

While many platelet-derived products have been investigated in the broader field of tissue engineering, the incorporation of these products into blood-contacting biomaterials to date has largely been limited to various forms of PL. Strategies for its use include simple immersion of a scaffold allowing for protein adsorption and/or absorption (150, 162) as well as emulsion or coaxial electrospinning (163–165). *In vitro*, incorporation of PRP into scaffolds has been shown to increase SMC adhesion, proliferation, infiltration, collagen production, macrophage chemotaxis, and adipose-derived stem cell chemotaxis and proliferation (150, 165, 166). Recently, addition of lysed PL to decellularized tissue-engineered vascular scaffolds implanted subcutaneously promoted a reparative response by elevating the M2/M1 ratio, reducing inflammation, and slowing the degradation process to allow for an extended period of recellularization and collagen deposition. This represents use of a platelet derivative to enhance the biocompatibility and regenerative properties of an implanted material intended for *in situ* vascular applications (167). Motivated by the need for small diameter tissue engineered vascular grafts, Li et al. utilized a PLLA, gelatin, and PL mixture in the electrospinning process to manufacture a vascular graft with the goal of increasing the rate of endothelialization after implantation (164). Grafts fabricated with PL released growth factors gradually over 25 days, increased the proliferation and migration of vascular endothelial cells and SMCs, as well as increased endothelial NO production and angiogenesis *in vitro*. In a rabbit common carotid artery replacement model, grafts made with PL displayed greater endothelization at the 1-week time point compared to control grafts. At 4 weeks, the vascular SMC layer thickness and eNOS expression by endothelial cells were significantly greater in grafts containing PL than the control group. Of note, rabbits were treated with aspirin and clopidogrel continuously throughout the implantation period. This dual strategy of utilizing a scaffold that slowly elutes PL along with pharmacological platelet inhibition demonstrates clear intention to control platelet-mediated signaling to optimize inflammation, neotissue formation, and remodeling.

## 10 Wielding the double-edged sword: understanding and modulating the FBR

*In situ* tissue engineering and regenerative medicine often involves the implantation of foreign material to generate autologous tissue. The immune system has been extensively optimized to protect an organism against invaders and the remarkable ability to determine self from non-self (e.g., cardiovascular biomaterials) through the FBR. In this context, the FBR is a double-edged sword that can either be helpful or harmful. The field now embraces a paradigm wherein modulation, but not suppression, of the immune response is necessary for biomaterial integration.

In this review, we establish that platelets are the first cellular responders to biomaterial implantation and are active contributors to the FBR to blood-contacting biomaterials. Their role extends beyond the traditional view as mere executors of hemostasis and thrombosis. Platelets are indispensable for biomaterial integration, shaping the material surface with a provisional matrix, recruiting and influencing other mediators of repair, and contributing to pro-regenerative signaling processes. Nevertheless, one must recognize the importance of selectively modulating platelet responses to optimize their pro-regenerative potential while preventing thrombotic complications. We contend that biomaterials currently in development should not only be evaluated for their propensity for thrombosis but also how platelets and their contents may be applied for optimizing tissue regeneration. This calls for a reevaluation of hemocompatibility. Standard testing for the blood compatibility of biomaterials established by the International Organization for Standardization includes a series of thrombogenicity tests. A material’s “failure” in such assays, which is often defined as exhibiting clinically relevant prothrombotic tendencies, should not be interpreted solely as a disqualifying feature. Instead, it should be reconsidered in the context of the platelet’s dual roles in both thrombosis and healing within the biomaterial environment. Such reframing enables the advancement of promising materials that might otherwise have been excluded from the translational pipeline, allowing them to progress on their own or combined with platelet-modulatory strategies.

The duration and degree of platelet modulation needed for each application will depend on the design of the cardiovascular device (i.e., biomaterial and pharmaceutical agent) and implementation strategy. There should also be dedicated studies comparing the advantages of local platelet modulation at the blood-biomaterial interface (e.g., surface coatings, localized drug delivery) versus the effects of systemic anti-platelet pharmaceuticals. These are critical questions that should guide future studies evaluating pharmacological modulation of platelets aimed at understanding the extent of modulation and its effects on biomaterial integration. Which platelet responses (i.e., aggregation, adhesion) are impacted? Do the therapies completely inhibit or merely blunt platelet function? What are the off-target effects? Do the benefits of platelet modification outweigh any introduced risks (e.g., bleeding)?

Overall, the role of platelets in neotissue formation and biomaterial integration has long been underappreciated. Recent studies indicating the pro-regenerative capacity of platelets warrant further attention for their benefits in the context of blood-contacting biomaterials. Systematically investigating the complete course of prosthetic healing and the role of platelets in these processes may provide the missing insights required to understand and ultimately guide the immune response. Armed with this knowledge, it becomes possible to deploy simple, off-the-shelf biomaterials while strategically modulating the platelet-mediated FBR to optimize clinical outcomes.
